# Distribution of Antimicrobial Resistance and Virulence Genes within the Prophage-Associated Regions in Nosocomial Pathogens

**DOI:** 10.1128/mSphere.00452-21

**Published:** 2021-07-07

**Authors:** Kohei Kondo, Mitsuoki Kawano, Motoyuki Sugai

**Affiliations:** a Antimicrobial Resistance Research Center, National Institute of Infectious Diseases, Higashi Murayama, Tokyo, Japan; b Department of Human Nutrition, Faculty of Contemporary Life Science, Chugokugakuen University, Kita-ku, Okayama, Japan; JMI Laboratories

**Keywords:** antimicrobial resistance, nosocomial pathogen, prophage, prophage-like element, virulence factors

## Abstract

Prophages are often involved in host survival strategies and contribute toward increasing the genetic diversity of the host genome. Prophages also drive horizontal propagation of various genes as vehicles. However, there are few retrospective studies contributing to the propagation of antimicrobial resistance (AMR) and virulence factor (VF) genes by prophage. We extracted the complete genome sequences of seven pathogens, including ESKAPE bacteria and Escherichia coli from a public database, and examined the distribution of both the AMR and VF genes in prophage-like regions. We found that the ratios of AMR and VF genes greatly varied among the seven species. More than 70% of Enterobacter cloacae strains had VF genes, but only 1.2% of Klebsiella pneumoniae strains had VF genes from prophages. AMR and VF genes are unlikely to exist together in the same prophage region except in *E. coli* and *Staphylococcus aureus*, and the distribution patterns of prophage types containing AMR genes are distinct from those of VF gene-carrying prophage types. AMR genes in the prophage were located near transposase and/or integrase. The prophage containing class 1 integrase possessed a significantly greater number of AMR genes than did prophages with no class 1 integrase. The results of this study present a comprehensive picture of AMR and VF genes present within, or close to, prophage-like elements and different prophage patterns between AMR- or VF-encoding prophage-like elements.

**IMPORTANCE** Although we believe phages play an important role in horizontal gene transfer in exchanging genetic material, we do not know the distribution of the antimicrobial resistance (AMR) and/or virulence factor (VF) genes in prophages. We collected different prophage elements from the complete genome sequences of seven species—Enterococcus faecium, Staphylococcus aureus, Klebsiella pneumoniae, Acinetobacter baumannii, Pseudomonas aeruginosa, Enterobacter cloacae, and Escherichia coli—and characterized the distribution of antimicrobial resistance and virulence genes located in the prophage region. While virulence genes in prophage were species specific, antimicrobial resistance genes in prophages were highly conserved in various species. An integron structure was detected within specific prophage regions such as P1-like prophage element. Maximum of 10 antimicrobial resistance genes were found in a single prophage region, suggesting that prophages act as a reservoir for antimicrobial resistance genes. The results of this study show the different characteristic structures between AMR- or VF-encoding prophages.

## INTRODUCTION

Antimicrobial resistance (AMR) is a global public health issue. In recent years, ESKAPE pathogens (Enterococcus faecium, Staphylococcus aureus, Klebsiella pneumoniae, Acinetobacter baumannii, Pseudomonas aeruginosa, and Enterobacter spp.) have become a threat since they are the leading cause of nosocomial infection and easily escape from authentic chemotherapy due to their antimicrobial-resistant phenotype, and many countries have faced difficulties in controlling these pathogens (1, 2). In Japan, Escherichia coli has replaced Staphylococcus aureus as the primary pathogen isolated from clinical samples in hospitals since 2018, and isolation of third-generation cephalosporin- or quinolone-resistant E. coli continues to increase in Japan (https://janis.mhlw.go.jp). Moreover, extended-spectrum of β-lactamase-producing E. coli is spreading worldwide (3). These ESKAPE and E. coli are major AMR pathogens in nosocomial settings.

A bacteriophage (phage) is a virus that infects bacteria. As soon as a phage adsorbs to the host’s cell wall, the phage genome is injected into the host cell. Temperate phages follow one of the two life cycles afterward: the lysogenic cycle or the lytic cycle. In the lysogenic cycle, the phage genome is integrated into the host chromosome, and it is called a prophage. In the lytic cycle, the prophage is induced to produce progeny phages in response to chemical or physical stressors (4, 5). Temperate phages or prophages are known to drive horizontal gene transfer (HGT) through transduction, but they also play an important role in increasing the genetic diversity of the host (6–10). Furthermore, defective prophages or prophage-like elements are stable in the host genome despite deleting most of the phage genes (6) and are known to increase host survival by conferring resistance against various stresses (11–13).

Plasmid conjugation has been well established as the major means of HGT of AMR genes (14), but recent studies have shed light on the role of phages in HGT of AMR genes since they are often encoded within the genome of the phage or prophage (15, 16). Metagenomic analysis revealed that AMR genes, such as β-lactamase, are found in the phage genome (17–19). A clinical study reported that phages harboring AMR genes were identified in samples from patients with cystic fibrosis (20). Costa et al. reported that many prophage regions within the A. baumannii genome possessed several AMR and virulence factor (VF) genes using a bioinformatics approach (21). These studies have implied that phages and prophages probably transduce AMR genes more frequently than expected. However, the relationship between prophages and AMR genes has not been fully explored.

Pathogenic or VF genes in prophages have been mainly studied in S. aureus (22, 23), E. coli (24, 25), Salmonella enterica (26), and *Vibrio* spp. (27, 28), and their pathogenicity is associated with VFs encoded by the prophages. Other reports revealed that the expression of the Shiga toxin in E. coli (29) and staphylokinase (*sak*) in S. aureus (30) depends on prophage induction. The studies mentioned above indicate that it is important to investigate the connection between host pathogenicity and their prophage to reveal their pathogenesis. However, very little is known of the relationship between virulence and prophages in the rapidly emerging multidrug-resistant bacteria, such as ESKAPE pathogens and E. coli.

This study aims to understand the distribution of AMR and VF genes encoded in prophages, including the intact region, prophage-like elements, and satellite prophages (31) comprised in the bacterial genome, and to discover their specific structural genomic features beyond the genera. We focus on seven clinically important AMR pathogens, including ESKAPE pathogens and E. coli, and analyzed their complete genomes deposited in a database to mine the prophage structure.

## RESULTS

### Comparison of host genome size and the number of prophages and prophage-like genomic islands.

To elucidate the distribution of AMR and VF genes encoded in the prophages within the genomes of ESKAPE bacteria, we collected complete genomes and RefSeq data of seven bacterial species (169 sequences of A. baumannii, 27 sequences of E. cloacae, 324 sequences of E. coli, 88 sequences of E. faecium, 408 sequences of K. pneumoniae, 183 sequences of P. aeruginosa, and 424 sequences of S. aureus) from GenBank (see Data Set S1 in the supplemental material). In this study, plasmid sequences were collected from 5 A. baumannii, 3 E. faecium, 14 K. pneumoniae, and 23 S. aureus isolates, while no plasmids were collected from E. coli, E. cloacae, and P. aeruginosa isolates; this was because the RefSeq plasmids of these species are not registered or are not selected. Subsequently, we detected prophages or prophage-like genomic islands in each genome using PHASTER (32). We investigated the correlation between the host genome size and the number of prophage elements present. The Pearson’s correlation coefficients (*R* values) of each species were 0.57 for A. baumannii, 0.51 for E. cloacae, 0.76 for E. coli, 0.67 for E. faecium, 0.51 for K. pneumoniae, 0.67 for P. aeruginosa, and 0.63 for S. aureus. The *R* values for all species were >0.5, indicating that the number of prophages positively correlated with the host genome size (see Fig. S1A).

10.1128/mSphere.00452-21.1 FIG S1 Comparison of genome length and the number of phage elements. (A) Correlation and scatter plot of genome length excluding plasmids and the number of prophages-like elements. Plasmid samples were excluded to avoiding genome length bias. RefSeq and the complete genome were collected from the NCBI, and we evaluated the correlation between each genome length and the number of prophages obtained from PHASTER. The *r* value indicates the Pearson’s correlation coefficient. (B) Box-and-whisker plot comparing the number of prophages for each species. The line within the box indicates the median. Welch’s *t* test was performed, and *P < *0.05 was considered significant. Download FIG S1, PDF file, 0.3 MB. Copyright © 2021 Kondo et al. 2021 Kondo et al. https://creativecommons.org/licenses/by/4.0/ This content is distributed under the terms of the Creative Commons Attribution 4.0 International license.

10.1128/mSphere.00452-21.5 DATA SET S1 List of host genomes and the number of prophages in each host genome. The number of prophages were counted by PHASTER. Genome size and definition were referenced to GenBank information. Download Data Set S1, XLSX file, 0.1 MB. Copyright © 2021 Kondo et al. 2021 Kondo et al. https://creativecommons.org/licenses/by/4.0/ This content is distributed under the terms of the Creative Commons Attribution 4.0 International license.

Next, we analyzed the number of prophages and prophage-like elements in each species. The number of prophages in E. coli was significantly higher than in other species (see Fig. S1B) (*P < *0.001): E. coli possessed a maximum of 24 prophages and prophage-related mobile elements (accession numbers CP027459 and CP024618), in which the prophages harboring the Shiga toxin genes *stx*_2A_ and *stx*_2B_ can be found.

Screening for prophage elements in plasmids yielded a few cases, as depicted in Fig. S2. Comparison of the numbers of prophages in plasmids indicated that most K. pneumoniae plasmids harbored at least one or more prophages or prophage-like elements, and some S. aureus plasmids possessed one prophage or prophage-like element. However, A. baumannii possessed no plasmids harboring prophage or prophage-like elements. The number of prophages harbored in K. pneumoniae plasmids was significantly higher than in A. baumannii and S. aureus plasmids (*P = *4.7 × 10^−5^ and *P = *0.0011, respectively) (see Fig. S1). AMR genes from prophage elements were encoded in plasmids found in K. pneumoniae (AP018748), whereas no VF genes were detected on plasmid-harboring prophages in the accession numbers used in this experiment.

10.1128/mSphere.00452-21.2 FIG S2 Comparison of the number of prophage-like elements present on the plasmid. The number of prophage regions existing on the plasmid was compared. Welch’s *t* test was performed, with *P < *0.05 considered significant. RefSeq plasmid sequences were not collected in E. cloacae and P. aeruginosa due to no plasmid being registered with the NCBI. In the case of E. coli, we did not select plasmid sequence because samples of E. coli were randomly selected from the NCBI. Download FIG S2, PDF file, 0.4 MB. Copyright © 2021 Kondo et al. 2021 Kondo et al. https://creativecommons.org/licenses/by/4.0/ This content is distributed under the terms of the Creative Commons Attribution 4.0 International license.

### Proportion of the number of AMR or VF genes to that of prophages in the host genome.

**FIG 1:**
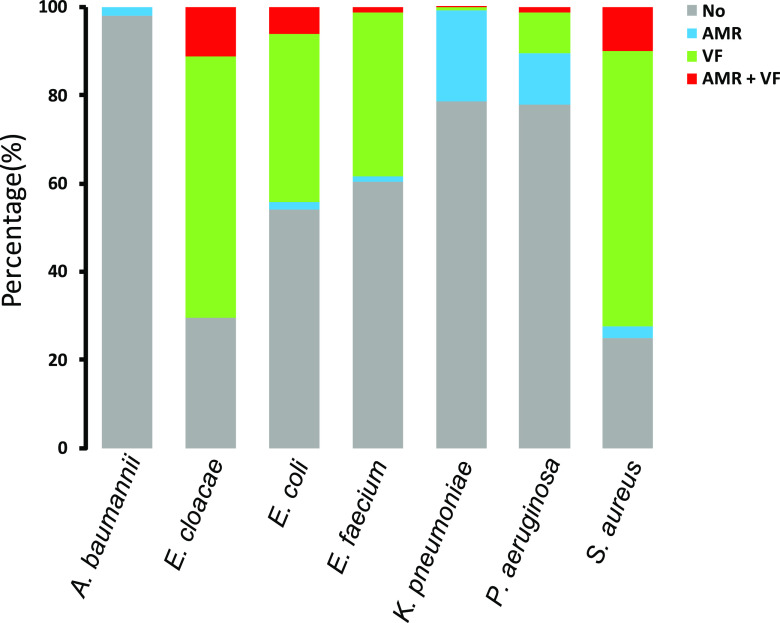
Proportion of the host genomes having prophages with AMR or VF genes. A bar chart shows the genome of each species that has AMR and/or VF gene-containing prophages. Blue represents strains with AMR genes, green represents strains having VF genes, and red represents the strains having both AMR and VF genes. Gray areas indicate strains with neither AMR nor VF genes.

We screened the prophage regions to exclude genomic islands without phage-related genes (see Materials and Methods) and examined the proportion of selected prophage elements encoding either AMR or VF genes. The proportions of the strains harboring prophages encoding VF genes were relatively high in E. cloacae, E. coli, E. faecium, and S. aureus (70.4, 44.2, 38.4, and 72.3%, respectively) (Fig. 1). In contrast, no prophages with VFs were detected in A. baumannii. Similarly, K. pneumoniae and P. aeruginosa also showed low proportions of VFs harbored in the corresponding prophages (1.2 and 10.5%, respectively) (Fig. 1).

Prophage regions encoding AMR genes were detected in all species. The proportions were as follows: 1.8% for A. baumannii, 11.1% for E. cloacae, 8.1% for E. coli, 2.4% for E. faecium, 20.9% for K. pneumoniae, 12.7% for P. aeruginosa, and 12.6% for S. aureus. The proportions of the host genome that contained both AMR and VF genes in the prophages were as follows: 11.1% for E. cloacae, 6.2% for E. coli, 1.2% for E. faecium, 0.3% for K. pneumoniae, 1.1% for P. aeruginosa, and 9.9% for S. aureus (Fig. 1). Overall, the differences in proportions for each species indicated that the ratio between AMR and VF genes differed greatly depending on the species.

### Characterization of prophage types encoding either AMR or VF genes.

**FIG 2:**
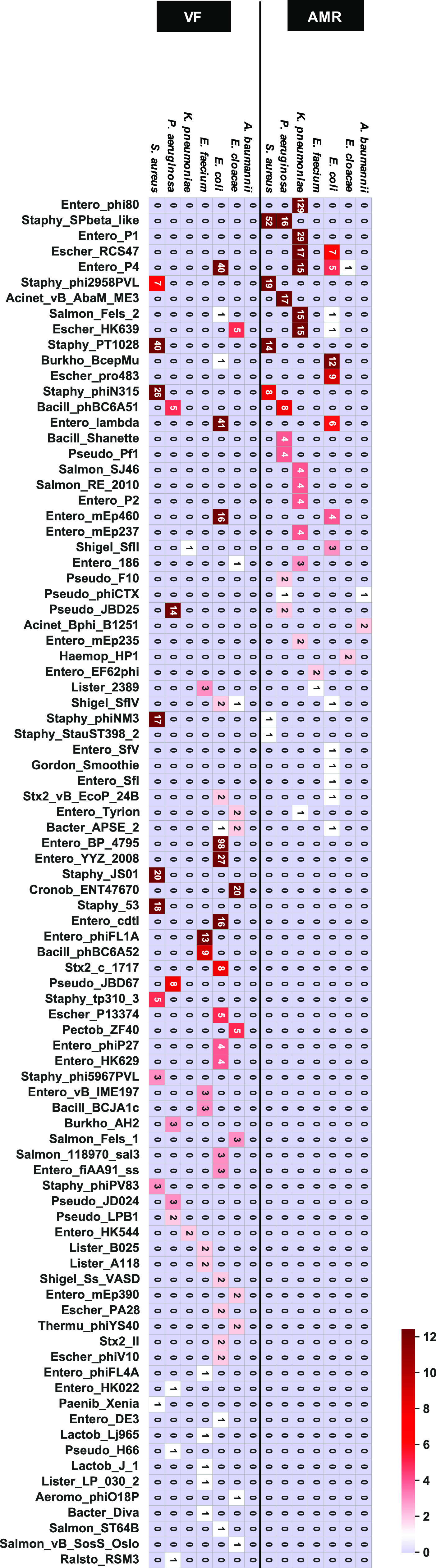
Types of prophages containing AMR and VF genes. Each prophage region that possessed AMR or VF genes was classified based on the most common phage in PHASTER. A heatmap shows the abundance of prophage type in each genus. The bottom shows the prophage types described in PHASTER. The numeric character in each cell represents the number of detected phage names.

We investigated the phage types integrated into the genome in each species. The name of each phage type was described using the most common phage indicated by the PHASTER database. The phage types harboring AMR gene(s) are listed in Fig. 2 and aligned according to the number of phage types harboring AMR gene(s) (Fig. 2). The number of phage types harboring AMR genes was 41 (Fig. 2). Notably, the major phage types carrying AMR gene(s), i.e., Escher_RCS47 (RCS47), Staphy_SPbeta_like, and Entero_P4 phages, were present in two or more species beyond the generic barrier. The P1 phage type, which is well known as a carrier of AMR genes, was detected in K. pneumoniae. In addition, the RCS47 phage is closely related to the P1 temperate phage and has newly acquired the insertion sequences (ISs) IS*26* and *blaSHV* (33). The RCS47 phage type was detected in E. cloacae, E. coli, and K. pneumoniae (Fig. 2). Our results suggest that P1-like phages are responsible for the transfer of AMR genes into the host genomes of bacteria from a broad range of genera.

In contrast to prophages containing AMR genes, we found that VF genes were widely distributed in a large number of phage types. There were 66 phage types; this number was 1.6-fold higher than the number of phage types harboring AMR genes. The prophage types carrying VF genes tended to be different from those harboring AMR genes, although some AMR gene-harboring prophage types were detected in VF-encoding prophages (Fig. 2). For instance, Entero_BP_4795 prophages, which are the most frequently detected phages carrying VF genes, were not detected in the prophages harboring AMR genes, whereas P4 phages were found to harbor AMR- and VF-encoding prophages (Fig. 2). Prophage types containing the VF genes hardly overlapped between different species. In other words, prophages containing VF genes are specific to each bacterial species. No prophages containing both AMR and VF genes are detected except in *E. coli* and *S. aureus* strains. Overall, these results indicated that AMR and VF genes rarely coexisted within the same prophage and that the distribution patterns of prophage types containing AMR genes were different from those of VF gene-carrying prophage types.

### Comparing complete genomes of prophages harboring AMR and VF genes.

**FIG 3:**
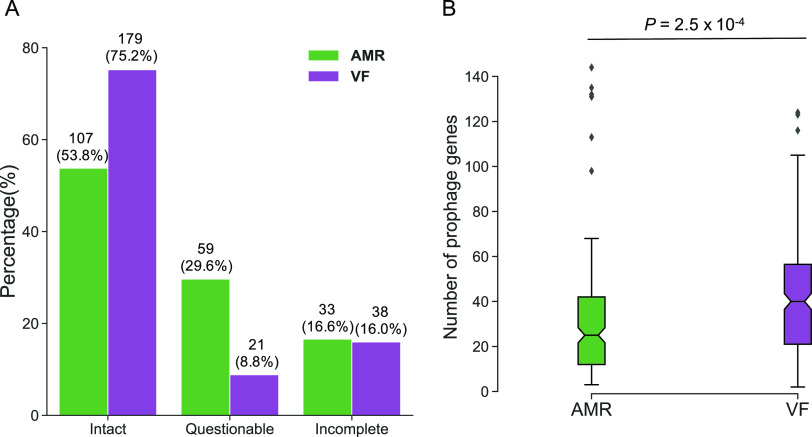
Analysis of prophage completeness harboring AMR or VF genes. (A) Each prophage that had AMR or VF genes was classified as incomplete, intact, or questionable depending on the length and their completeness. The green bars show the percentage of prophages with AMR genes, the purple bars show the percentage of prophages with VF genes. The percentages regarded all AMR- or VF-encoding prophages as 100%, and the numerical character below the percentage indicates the number of samples in each classification. (B) The prophage genes were annotated using BLASTp. The genes involved in integrase, transposase, and AMR/VF genes were excluded, and the numbers of prophage genes in each region were counted. Welch’s *t* test was performed, with *P < *0.05 considered significant.

We next compared the completeness of prophage-encoding regions carrying AMR and VF genes. The prophage regions were classified using criteria from PHASTER as either intact, questionable, or incomplete. As shown in Fig. 3, percentages of prophages with AMR genes were 16.6, 29.6, and 53.8% for incomplete, questionable, and intact ones, respectively. Prophages carrying VF genes were mostly intact (75.2%), which indicated that the prophage retained most of its region, whereas 16.0 and 8.8% were incomplete and questionable phages, respectively (Fig. 3A). In contrast to prophages carrying AMR genes, those carrying VF genes often encoded proteins that were crucial for the structure of the phage, such as the head, tail, and baseplate (see Data Sets S4 and S5). Evidently, all prophages encoding Shiga toxins in E. coli were intact and contained phage structural proteins (see Data Sets S4 and S5). Next, we compared the number of all phage-related genes, except for transposase-, integron integrase-, and AMR/VF-encoding genes, in each prophage region. The number of prophage-related genes in VF-encoded prophages was more than that of AMR genes (*P = *2.5 × 10^−4^) (Fig. 3B). These results imply that AMR-encoding prophages are prone to be inactivated and degraded and thus are likely to become defective prophages.

### Characterization of VF genes in prophages.

**FIG 4:**
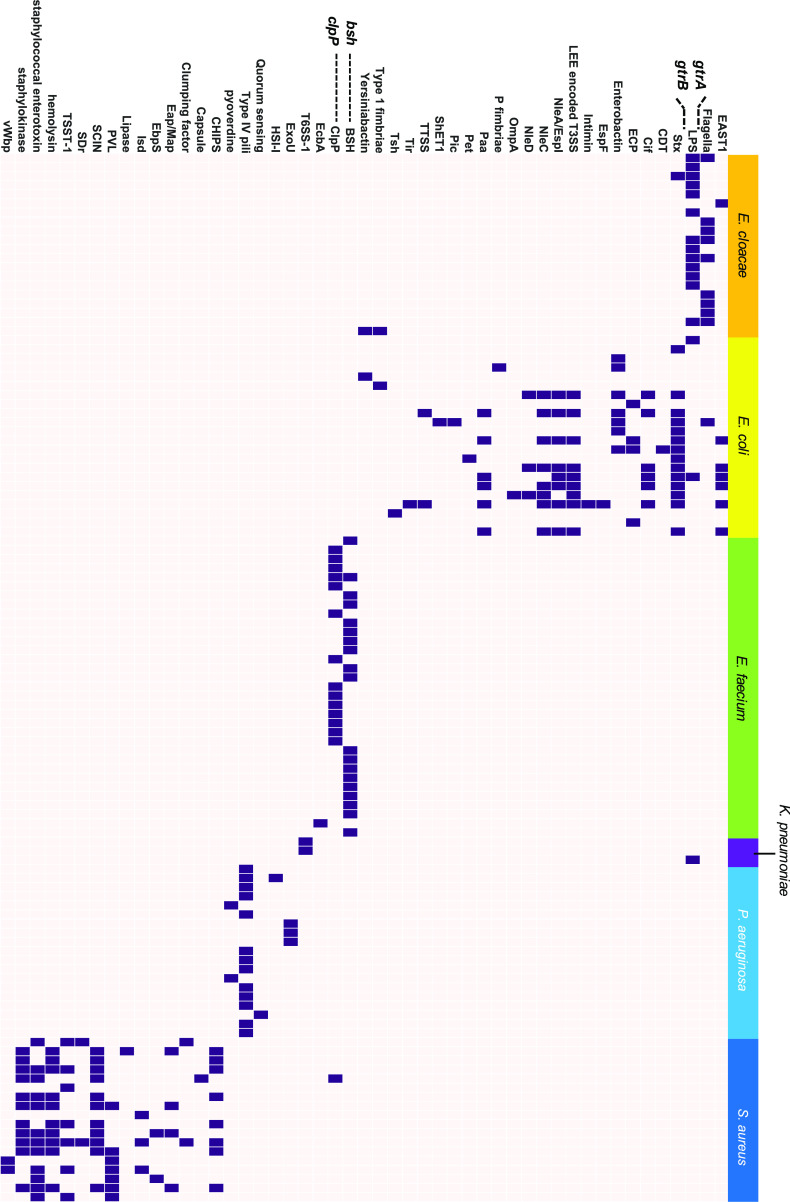
Distribution of VF genes in the prophage regions. DNA sequences of the prophage regions extracted from PHASTER were examined for the presence or absence of various VF genes using the VFDB. Color in the heatmap indicates whether genes are present or not (purple square, presence, white square; absence). VF genes in S. aureus and E. coli are shown as representative strains. All VF genes harbored by prophages are listed in Data Set S3. EAST, heat-stable enterotoxin 1; CDT, cytolethal distending toxin; Cif, cyclomodulin; LEE, locus of enterocyte effacement; TTSS, type 3 secretion system; CHIPS, chemotaxis-inhibiting protein; PVL, Panton-Valentine leucocidin; SCIN, staphylococcal complement inhibiter; TSST-1, toxic shock syndrome toxin 1.

To further investigate VF genes in prophage elements, VF genes for each accession number were examined using the Virulence Factors Database (VFDB) from ABRicate, and the presence of VF genes in prophages was visualized in the matrix (Fig. 4).

Prevalence of prophage-encoded VF genes was unique to each species (Fig. 4). For example, *gtrA* and *gtrB*, which encode bactoprenol-linked glucose translocase/flippase, were detected in 65.0% of E. cloacae strains, in one K. pneumoniae strain, and in one E. coli strain, but not in strains of other species (Fig. 4). Bacteriophage ENT47670 (accession number NC_019927) contained *gtrA* and *gtrB*. Interestingly, intact ENT47670 prophage, which also contained structural phage proteins, was detected in the prophage region where *gtrA* and *gtrB* were harbored (see Data Sets S3 and S5). This result indicates that *gtrA* and *gtrB* of the prophage region in E. cloacae were acquired through the transduction of ENT47670 or ENT47670-like phages (34).

E. faecium strains had two specific genes in the prophage regions—*bsh* encoding a bile salt hydrolase and *clpP* encoding an ATP-dependent protease—but other species did not (Fig. 4). We mapped the prophage region harboring *bsh* and *clpP* in E. faecium using BRIG (35) (see Fig. S3). We found that these genes were generally located at or near the gaps in the genome and were scattered at various locations in the chromosome. It has been assumed that *bsh* genes in E. faecium have been transferred via HGT because the G+C content of *bsh* gene is different from the genomic G+C content of this organism (36). Our results indicate that temperate phage-mediated transduction is one of the factors responsible for the transfer of *bsh* genes in E. faecium.

10.1128/mSphere.00452-21.3 FIG S3 Mapping of *bsh* and *clpP* prophage encoding in E. faecium. The red circles (inner circle) indicate the genome that exists *bsh* genes in prophage region, and the green color circles indicate the genome that does not encode *bsh* in the prophage region. The red arc indicates the prophage region encoding *bsh*, and the black arc indicates *clpP*. The gap shows that has less homology (<50%) with the original genome. The figure was created using BRIG. Download FIG S3, PDF file, 0.1 MB. Copyright © 2021 Kondo et al. 2021 Kondo et al. https://creativecommons.org/licenses/by/4.0/ This content is distributed under the terms of the Creative Commons Attribution 4.0 International license.

Classification of VF genes located in prophages revealed using VFDB keywords showed that each species possesses functionally unique VF genes (see Fig. S4).

10.1128/mSphere.00452-21.4 FIG S4 Classification of VF genes encoded in prophage. Each kind of VF gene was classified based on the keywords described in the VFDB. VF genes harbored by prophages were not found in A. baumannii. The numerical value in the heat map indicates the percentage (%) classified into keywords for each strain. Download FIG S4, PDF file, 0.2 MB. Copyright © 2021 Kondo et al. 2021 Kondo et al. https://creativecommons.org/licenses/by/4.0/ This content is distributed under the terms of the Creative Commons Attribution 4.0 International license.

### Characterization of AMR genes encoded by or nearby prophage elements.

**FIG 5:**
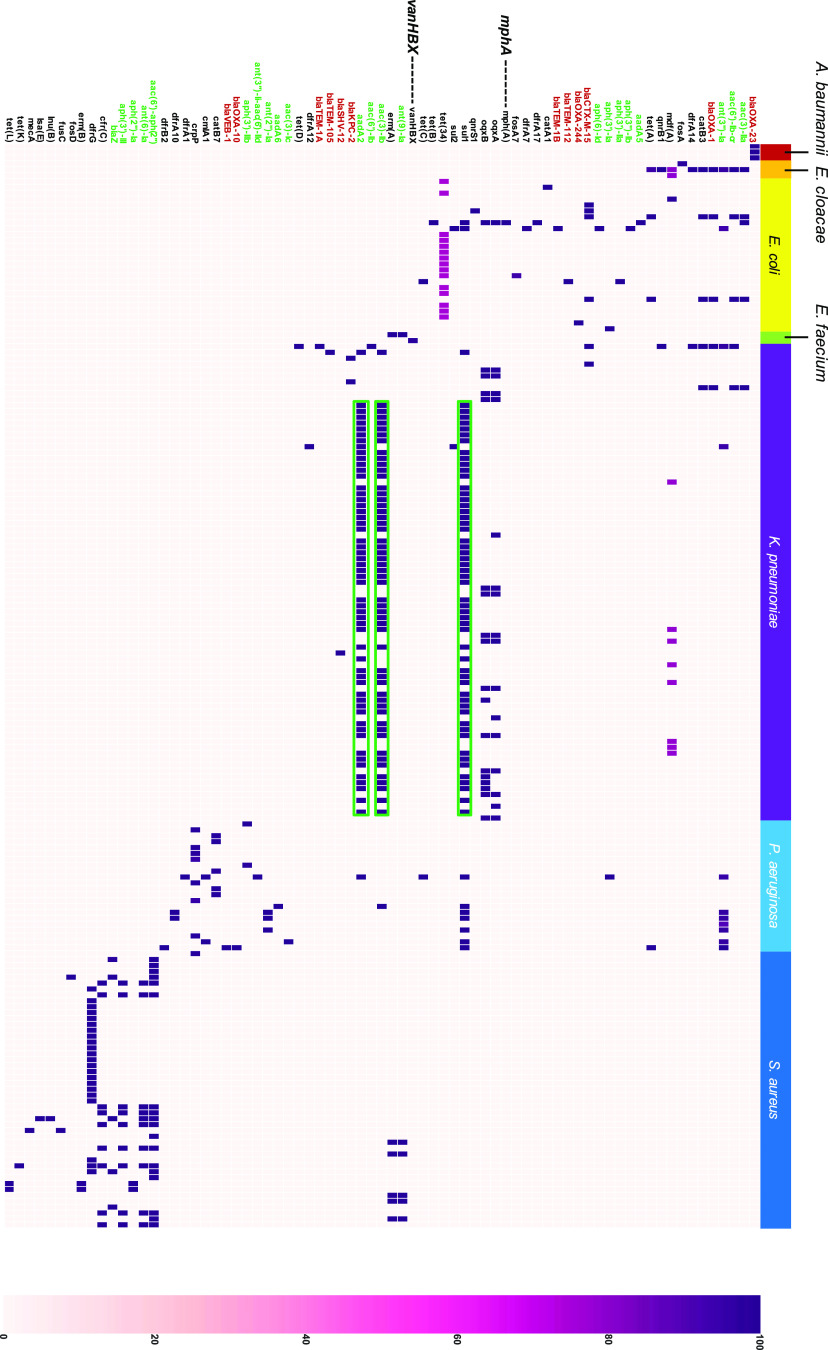
Distribution of AMR genes in the prophage region. We investigated the AMR genes in prophage regions extracted from PHASTER. AMR genes were detected using the ResFinder database. The color density of the heatmap represents the identity (%) of each gene. Green frame indicates the AMR genes cassette array detected in the K. pneumoniae. AMR genes, highlighted in green and red, show resistant genes against aminoglycoside and β-lactam, respectively. Magnified AMR gene names represent AMR genes described in Results.

To investigate AMR gene distribution in the prophage elements of each strain in detail, AMR genes in the prophage region for each accession number were extracted using the ResFinder database from ABRicate and classified based on predicted substrates and mutation locations (see Materials and Methods). Aminoglycoside-modifying enzyme-associated gene(s) and β-lactamase are widely distributed among the seven bacterial species studied here (Fig. 5).

Apart from the genes encoding aminoglycoside-modifying enzymes and β-lactamase, which have been reported in various species, few species-specific AMR genes were also detected. For instance, the intact pro483 type prophage in E. coli (CP019256) harbored two copies of *mphA*, encoding macrolide 2′-phosphotransferase I. *vanHBX*, conferring resistance to vancomycin, was detected in only E. faecium, and *vanHBX* was harbored in “intact” prophages with the genes encoding some structural proteins (see Data Sets S2 and S4). These data suggested that a part of *van* family of genes in vancomycin-resistant E. faecium were acquired via phage transduction.

**FIG 6:**
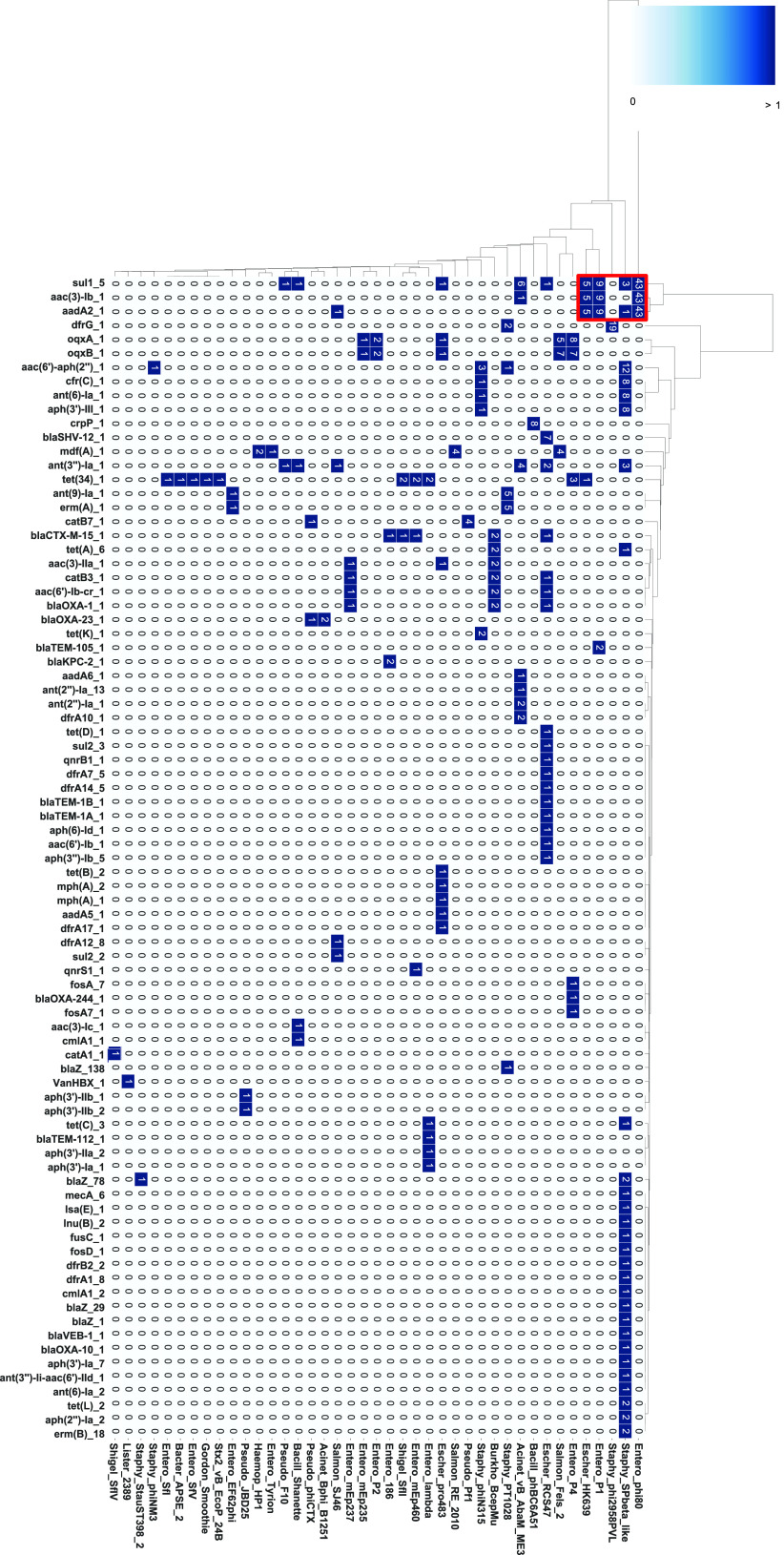
Cluster analysis of AMR gene-encoding prophages. Each prophage type included in all bacterial species used in this study was clustered under the same prophage name and visualized via a heatmap. The figure was created using seaborn, which is a Python module, and cluster method, which is a hierarchical clustering method (single linkage method). The numerical characters in cells mean the number of AMR genes in the indicated prophage. The red frames indicate the examples of cassette arrays of AMR genes in the prophage region.

AMR gene cassettes were widely detected in various species. For instance, K. pneumoniae contained a cassette array of AMR genes, including *sul1*, *aadA*, and *aacA* (Fig. 5). To detect the combination or the gene cassette of AMR genes harbored by prophage elements, we classified and clustered AMR gene cassettes for each prophage type (Fig. 6). Prophage regions from 43 Entero_phi80, 9 Entero_P1, and 5 Escher_HK639 harbored a set of AMR genes, including *sul1_5*, *aac(3)_lb_1* and *aadA2_1* (Fig. 6; see also Data Set S2). Furthermore, another cassette array containing *aac(6′)-aph(2″)*, *ant(6)-Ia*, *aph(3′)-III*, and *cfr*(*C*) was detected in more than eight Staphy_SPbeta_like prophage regions and one Staphy_phiN315. Since these AMR gene combinations detected on prophage regions resemble the integron cassette array, we tried to identify the integrons in these prophage regions using the INTEGRALL database. We found that specific prophage regions in K. pneumoniae, E. coli, and P. aeruginosa possessed class 1 integrase and AMR gene cassette arrays (see Data Set S2). Therefore, we considered these characteristic regions containing AMR genes cassette arrays as integron cassette arrays (integron-associated prophage structures). Although the structure of integron-associated prophages has been hardly reported, our results clearly reveal that specific prophage elements, including those in P1-like phages, are preserved in the genomes of ESKAPE bacteria.

10.1128/mSphere.00452-21.6 DATA SET S2 Information on AMR genes within the prophage in each strain genome. Most common phage and completeness were referenced to PHSATER. The Int column represents the type of integrase. Integron number (In number) was decided depending on the array of AMR gene cassette according to INTEGRALL. The integron region was confirmed whether each integron is located in the prophage or not. If the integron is integrated in the prophage region, we described this as “Yes” in the cassette array in the prophage column. Prophage regions shaded red possess both AMR and VF genes. Download Data Set S2, XLSX file, 0.03 MB. Copyright © 2021 Kondo et al. 2021 Kondo et al. https://creativecommons.org/licenses/by/4.0/ This content is distributed under the terms of the Creative Commons Attribution 4.0 International license.

**FIG 7:**
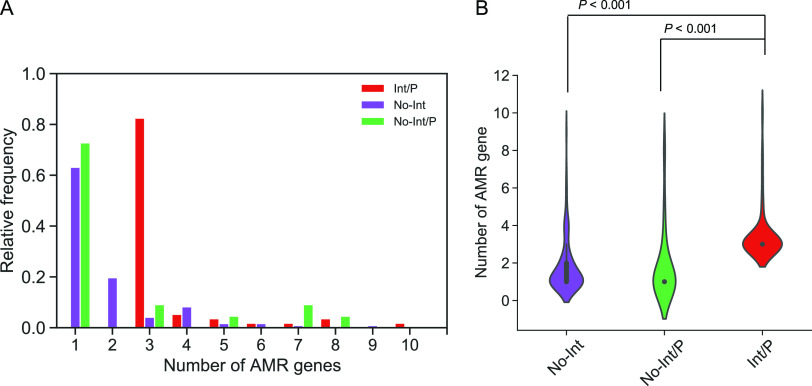
Comparison of the number of AMR genes with and without an integron. No-Int is a strain that does not have an integron, No-Int/P represents that the integron is located out of the prophage region, and Int/P is a strain that has an integron in the prophage. Integron within a prophage was classified according to whether it has class 1 integrase or not. (A) A histogram shows the relative frequency of each number of AMR genes in each classification. (B) A boxplot shows the number of AMR genes in each classification. The notch of the boxplot represents the median, and Welch’s *t* test was performed, with *P < *0.05 considered significant.

Next, we wondered whether the prophage region containing integrons had a higher number of AMR genes than regions without integrons (Fig. 7). We examined the number of AMR genes in phage regions carrying integrons (Int/P, red), in those without integrons (No-Int, green), and in strains where integrons were present somewhere other than the prophage region (No-Int/P, blue).

All the phage regions carrying an integron possessed three or more AMR genes, whereas 82.7% of the phage regions without an integron possessed fewer than three AMR genes (Fig. 7A). These results indicated that the number of AMR genes were significantly higher in prophages with integrons than in other groups (*P < *0.001) (Fig. 7B).

### Structural features of prophage elements containing AMR genes.

**FIG 8:**
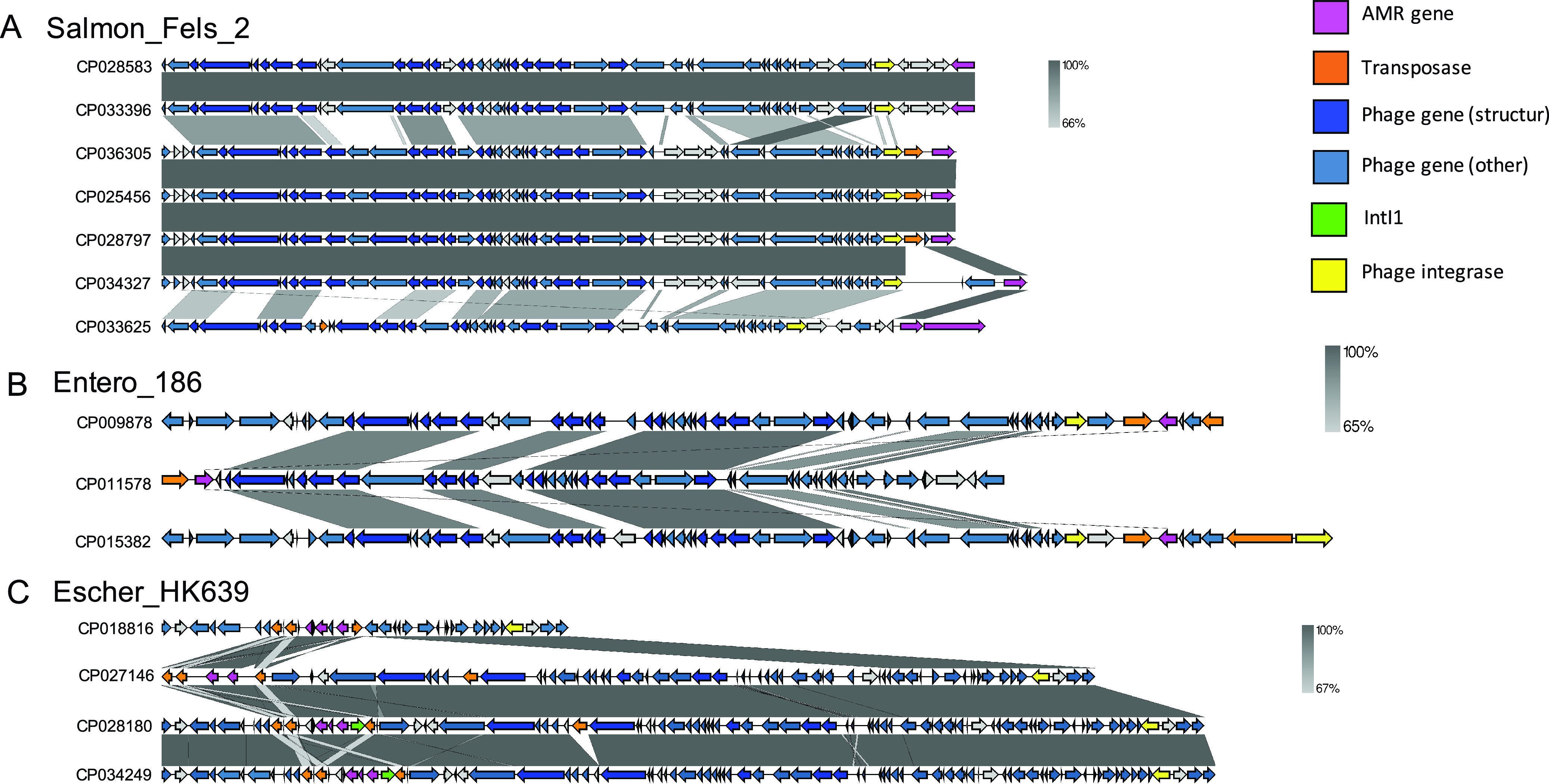
Comparative analysis of sequences and positional characteristics of AMR genes located in prophages. Representative prophage type names were selected based on genome completeness. Prophage sequences were defined via the attachment sites (*attL* and *attR*). Three representative prophages—Salmon_Fels_2 prophage (A), Entero_186 (B), and Escher_HK639 (C)—were selected and visualized to show the structural feature of prophage and AMR genes. The sequences of each prophage were selected at random, and gray-shaded regions represent sequence similarity. IntI1 shows the class 1 integrase, which is indicated by the green arrow, and phage-derived integrase is indicated by the yellow arrow. AMR genes are shown in magenta. Phage genes were shown by blue (structural genes) and steel blue (other than structural genes) arrow. The figures were drawn using Easyfig 2.2.2.

We briefly overviewed the structural features of the prophage region and its association with AMR gene(s) by showing representative prophage sequences (Fig. 8). The prophage regions within the attachment sites (*attL* and *attR*) are shown in Fig. 8. AMR genes in intact prophage regions existed either between or near integrase and/or transposase genes (Fig. 8A and B). Furthermore, AMR genes were located at the end of the prophage region, whereas the central position in the prophage region often encoded essential phage genes, especially structural proteins. Although the intact phages Salmon_Fels_2 and Entero_186 do not harbor class 1 integron integrase, phage-derived integrase was present near to AMR gene(s) (Fig. 8A and B). Next, we visualized the prophage regions from Escher_HK639, which were described as intact or questionable phages using PHASTER (Fig. 8B). The prophage region from Escher_HK639 contained in K. pneumoniae (accession number CP018816) is described as one from a questionable phage; the other three HK639 phages (shown in Fig. 8C) are intact phages, which also contain the structural proteins. Although most phage-related genes in HK639 harbored by CP018816 are degraded, BLAST comparison (based on nucleotide sequences) showed that the sequences of these genes are identical to the distal sequences of other HK639 regions. This result implies that the HK639-like phages are excised from the CP018816 genome, but the end of the HK639-like phage sequence are left in the host genome. AMR genes are located at integron structures at the end of the HK639 prophage region (integron-associated prophage structures), indicating that the chromosomal locus where the prophage has integrated can accommodate other elements, such as transposons and AMR genes; this locus can be transferred to other bacteria via specialized or generalized transduction (18).

## DISCUSSION

In this study, we collected the prophage elements from a total of 1,623 complete genomes of nosocomial AMR pathogens deposited in a public database and characterized AMR and VF genes harbored. Due to multidrug resistance, ESKAPE bacteria have become a threat to global health, especially in elderly patients. HGT agents such as plasmids, prophages, and transposons often carry AMR and VF genes and move from bacteria to bacteria. Phage or prophage-carrying AMR genes are reported to be minor HGT agents (37, 38). In recent years, however, several studies have reported that AMR genes are present in the phage or prophage region (39–42), implying that they are associated with the prophage region more than expected. Nevertheless, there have been few reports comprehensively identifying and analyzing AMR/VF genes located in the prophage region of nosocomial AMR pathogens.

Our study demonstrated that the proportion of bacteria carrying AMR genes on prophage-like elements reached up to 21% (Fig. 1). Furthermore, our analysis showed that the integron-associated prophage structure is often located at the side of AMR genes (Fig. 8). Our results suggest that the number of AMR genes encoded by integron-associated prophages was higher than that of those encoded in the genome of phage particles and that AMR genes were preferentially accumulated in the prophage sequence and were stably inherited in the host genome (6). To date, plasmids are the most well-studied HGT agent. Nonetheless, we did not compare the AMR gene richness between plasmids and prophage regions. Several prophage-like elements encoding AMR genes were inserted and shared AMR genes in a plasmid; thus, we solely focused on the prevalence of AMR genes encoded by prophage regions to shed light on the previously overlooked contributions of prophages in AMR gene propagation.

Prophage induction is triggered mainly by DNA damage via SOS response, and the host is lysed by the phage lysin. We found that 46.2% of AMR gene-harboring prophages were defective, whereas intact prophages were dominant among VF gene-harboring prophages (75.2%) (Fig. 3). A previous study has shown that prophages confer antibiotic resistance to the host and result in an increase of host viability (43). In other words, the host takes the advantage of AMR gene products of prophages for survival. A recent study has reported that the induction frequency of prophage carrying AMR genes decreases under the presence of antibiotics (44). As a result, prophages tend to become defective more frequently and are inherited in the host genome (45). In contrast, it has been reported that VF genes encoded in prophage can contribute to increasing phage infectivity by increasing the burst size and the latent period (46). In addition, prophages often harbor the genes involved in superinfection exclusion, a phenomenon in which phage or prophage prevents infection by other phages (47, 48), and such genes are associated with host virulence (46). Therefore, we speculated that VF genes encoded in prophages have presumably more benefits for the prophage rather than for the bacteria, and the selective pressure of becoming defective is hardly caused in VF gene-encoding prophage. As a result, prophages containing VF genes tend to remain “intact” with phage structural genes.

Our results showed that most VF genes on prophage element were species specific (Fig. 5), presumably because the mechanism of virulence to the host, such as entry into the mammalian body (cell), and the toxicity differs depending on the species. Thus, each VF gene, which is responsible for virulence, could be detected in a species-specific manner. In contrast, modes of action of antimicrobials are common among bacterial species, and AMR genes are mobilized among bacterial genera through horizontal transfer; thus, similar AMR genes encoded in prophage elements are conserved in various bacterial species.

We found that many prophages harboring AMR genes possessed an integron structure, and the numbers of AMR genes in integron-harboring prophages were significantly higher than in prophage regions without an integron (Fig. 8). Recently, P1-like phage element sequences with integron structures have been detected in several plasmids of pathogenic E. coli (41) and P1-like phage group has been found at plasmids in various bacterial phyla (49). However, whether bacterial genomes harbor the integron-associated prophage structures has not yet been ascertained. Our results clearly showed that such a structure was detected in the genomes of K. pneumoniae, P. aeruginosa, and E. coli (Fig. 8; see also Data Set S2). Even if prophages carrying AMR genes do not possess integron structures, recombination elements, such as transposase and phage site-specific integrase genes, are located close to AMR genes (Fig. 8). Indeed, the Mu-like phage is present on a plasmid, and transposon Tn*21* is encoded within the phage region of the plasmid, which also includes AMR genes (50). Although our results do not directly prove the hypothesis that AMR genes adjacent to prophages are acquired by phage transduction, prophage-related recombination genes and integrons harbored by prophages probably accommodate the AMR genes. Our analysis suggests that unlike prophages containing AMR-encoding genes, prophages containing VF-encoding genes are not likely to possess recombination-related genes near to VF genes.

It has been known that genes encoding aminoglycoside modification enzymes and β-lactamase are widely conserved among various species (51, 52). In this study, aminoglycoside modification enzymes and β-lactamase were detected in prophages and prophage-like elements of almost all genera, in accordance with previous studies (51, 52) (Fig. 5). This result suggests that phages were involved in the HGT of these highly distributed AMR genes. In fact, some AMR genes (β-lactamase-encoding genes) that are associated with phage-related mobile elements are widely distributed across various members of the family *Enterobacteriaceae* (53).

This study has some limitations. The sample number is uneven between each species. For example, the sample number of E. cloacae was 27, while that of S. aureus was 408. Since most of the complete genomes of E. coli are enterohemorrhagic *E. coli* (EHEC) genomes, Stx (classified as “toxin” in Fig. S3) and type III secretion systems were detected using the VFDB, in agreement with previous reports (54, 55). Therefore, the distribution of VF genes and the percentage of toxin and type III system harbored by prophages may have been influenced by the E. coli EHEC data. To analyze detailed and precise prophage sequence structure, we utilized a highly accurate prophage sequence using the National Center for Biotechnology Information (NCBI) RefSeq (56). Because most of these samples registered in RefSeq are isolated from the clinical settings or represent pathogenic bacteria, these samples may be associated with bias. Further analyses, such as metagenomic approaches, are required for more equitable assessments of the role of HGT in phages. In fact, metagenomic studies have revealed that phage fractions from various samples contain AMR genes (15, 57).

Overall, we comprehensively detected AMR and VF genes in a wide range of strains, and our results will shed light on the important roles of phages as reservoirs and factors that transfer AMR/VF genes. Further research is needed to elucidate the number of AMR and VF genes that are transferred to other strains via transduction in a clinical and natural environment.

## MATERIALS AND METHODS

### Data collection and prophage region detection.

We compiled complete genomes and RefSeq data of seven bacterial species (169 sequences of A. baumannii, 27 sequences of E. cloacae, 324 sequences of E. coli, 88 sequences of E. faecium, 408 sequences of K. pneumoniae, 183 sequences of P. aeruginosa, and 424 sequences of S. aureus) from GenBank using Biopython version 1.76 (58). Complete genome data sets were collected in December 2019. The genome size for each strain was referred to as the value described in GenBank (see Data Set S1). Since E. coli had a large number of registrations at the NCBI, GenBank accession numbers were randomly selected so that the sample size would not increase. To detect prophages and prophage-like elements (28) for each strain, we used a custom application programming interface from PHASTER (32). Prophage names were identified using the most common phage species indicated by PHASTER. In addition, PHASTER was also used to classify prophages as either intact, questionable, or incomplete based on the length of the phage region and the number of phage-derived genes (see Data Sets S4 and S5).

### Detection of AMR and VF genes.

AMR and VF genes encoded by the prophage sequences were extracted using ABRicate version 1.0.1 (https://github.com/tseemann/abricate) under default settings. The ResFinder database (59) was used to detect AMR genes, and the Virulence Factors Database (VFDB) (60) was used to detect VF genes. VFs were classified based on VFDB keywords, and redundant (similar) keywords were summarized into short words (e.g., “adhesion,” “apoptosis and adherence,” and “invasive” keywords were integrated into “adherence,” and toxin-based genes were integrated into “toxin”). The same gene with a few, different mutations were distinguished as an accession number were assigned to each gene containing the respective mutation. Each of the genes was also assigned a “_X” suffix.

### Selection of prophage regions harboring AMR and VF genes.

Prophage element regions comprising AMR/VF genes were further selected to eliminate genomic islands such as ISs and integrons without any phage-related genes. The open reading frames (ORFs) of prophage regions containing AMR and VF genes were selected using Prokka (61) with default settings; the functional annotation of ORFs were performed using local BLASTp (62). The number of prophage-related genes in each prophage region were counted. The phage-related genes did not include integron integrase, transposase, and any AMR/VF genes. If prophage regions have more than four prophage-related genes whose E value is less than 2E–20, these regions were considered true prophage regions.

### Integron analysis.

Integrons and antibiotic cassette gene arrays in prophages were detected using INTEGRALL (63). Ambiguous integrases encoded in prophage region were examined using BLASTP (protein-protein BLAST). If the amino acid identity was more than 80%, it was considered a class 1 integrase. Numbers were assigned based on the order of the gene cassette, and we referred to the number described in INTEGRALL. We classified the results into three groups, depending on the type of integron arrangement, as follows: (i) prophage region with a class 1 integrase (Int/P); (ii) prophage region without a class 1 integrase (No-Int); and (iii) integrase present but it did not exist in the prophage region (No-Int/P).

### Prophage elements and other data visualization.

Prophage elements encoding *bsh* and *clpP* were visualized using BLAST Ring Image Generator (BRIG) version 0.95 (35). All BRIG parameters were set to default. Other prophage-related regions containing AMR genes were visualized using Easyfig version 2.2.2 (64). The thresholds of BLAST hits in Easyfig were analyzed using the default value. To visualize, we selected Escher_HK639, Entero_186, and Salmon_Fels_2, which were regarded as “intact” phages, encoding a phage structure protein. Alternatively, we selected Acineto_vB_AbaM_ME3, Entero_phi80, and Escher_RCS47, which were considered “incomplete” or “questionable” in PHASTER. The host accession number used for the analysis had three to five strains selected at random for each prophage. Other data were analyzed and visualized using Python 3.7.6 (Python Software Foundation, https://www.python.org), Matplotlib version 3.1.3, and Seaborn version 0.1.0.

### Statistical analysis.

Pearson R correlation was calculated using the default jointplot function in Seaborn. All statistical analyses were conducted using a two-sided Welch’s *t* test with Python version 3.7.6 and SciPy Module version 1.4.1, and *P < *0.05 was considered a significant difference.

### Data availability.

This study was performed using complete genomes registered on the NCBI. All accession numbers are listed in Data Set S1. All information on AMR and VF genes detected in this study are described in Data Sets S2 and S3. The information on integron is summarized in Data Set S2. Information on specific phage genes and the presence of attachment sites (*attL* and *attR*) in each prophage region is presented in Data Sets S4 and S5.

10.1128/mSphere.00452-21.7 DATA SET S3 Information on VF genes within the prophage in each strain genome. The most common phage and completeness were referenced to PHSATER. Prophage regions shaded red possess both AMR and VF genes. Download Data Set S3, XLSX file, 0.03 MB. Copyright © 2021 Kondo et al. 2021 Kondo et al. https://creativecommons.org/licenses/by/4.0/ This content is distributed under the terms of the Creative Commons Attribution 4.0 International license.

10.1128/mSphere.00452-21.8 DATA SET S4 Number of AMR genes encoded in each prophage region and the completeness of each prophage. Specific phage keywords indicate the phage-related genes detected in each prophage region according to PHASTER. Attachment site columns represent the presence of *attL* and *attR* sites. Download Data Set S4, XLSX file, 0.02 MB. Copyright © 2021 Kondo et al. 2021 Kondo et al. https://creativecommons.org/licenses/by/4.0/ This content is distributed under the terms of the Creative Commons Attribution 4.0 International license.

10.1128/mSphere.00452-21.9 DATA SET S5 Number of VF genes encoded in each prophage region and the completeness of each prophage. Specific phage keywords indicate the phage-related genes detected in each prophage region. Download Data Set S5, XLSX file, 0.02 MB. Copyright © 2021 Kondo et al. 2021 Kondo et al. https://creativecommons.org/licenses/by/4.0/ This content is distributed under the terms of the Creative Commons Attribution 4.0 International license.
