# Supplementary figures and images for: Distribution of Antimicrobial Resistance and Virulence Genes within the Prophage-Associated Regions in Nosocomial Pathogens
Source: mSphere. 2021 Jul 7;6(4):e00452-21. doi: 10.1128/mSphere.00452-21 (PMC8386436; doi:10.1128/mSphere.00452-21)

A

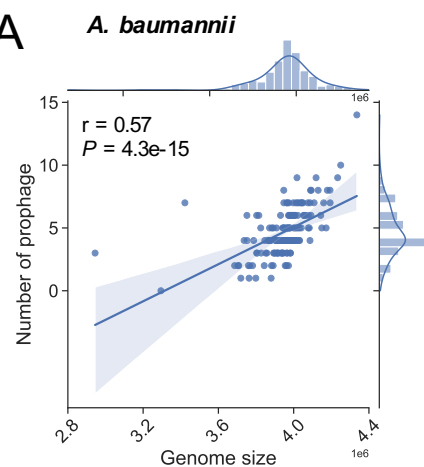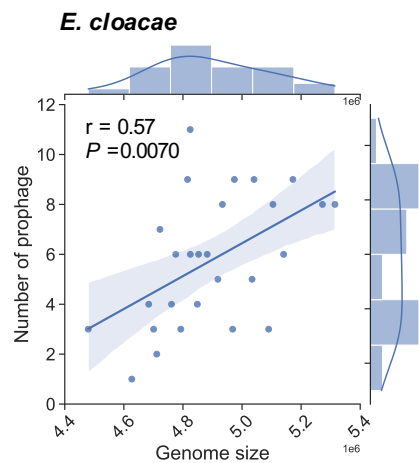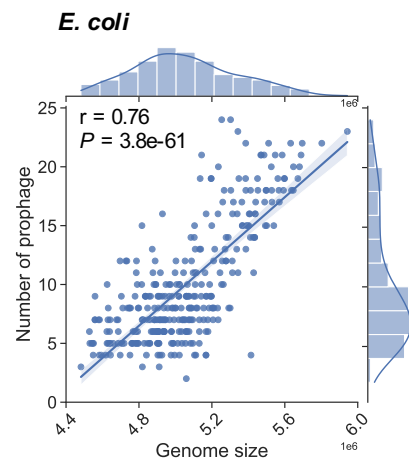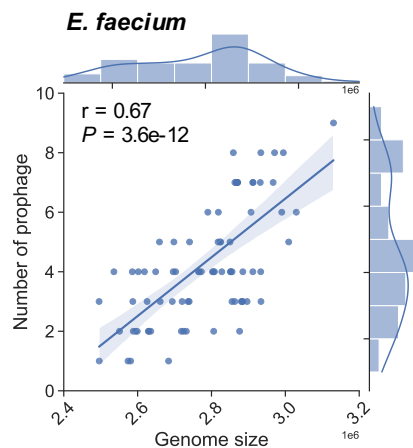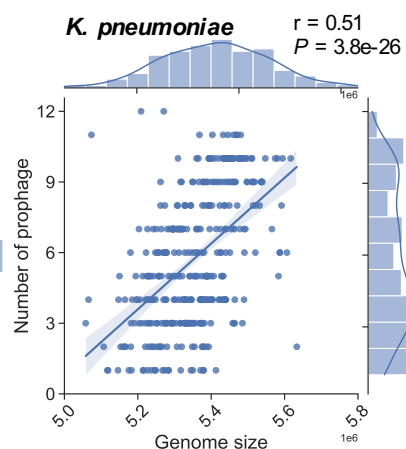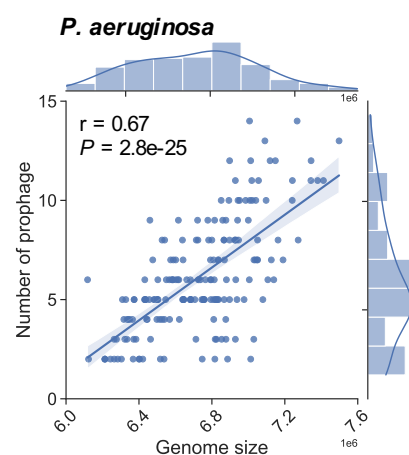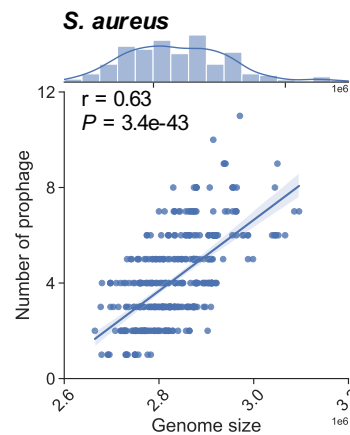

B

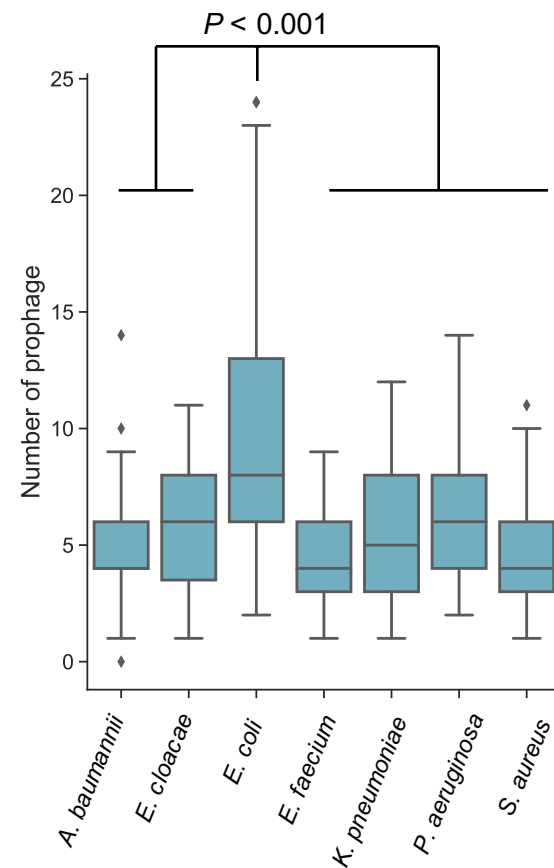

Supplement: FIG S1 [file msphere.00452-21-sf001.pdf]

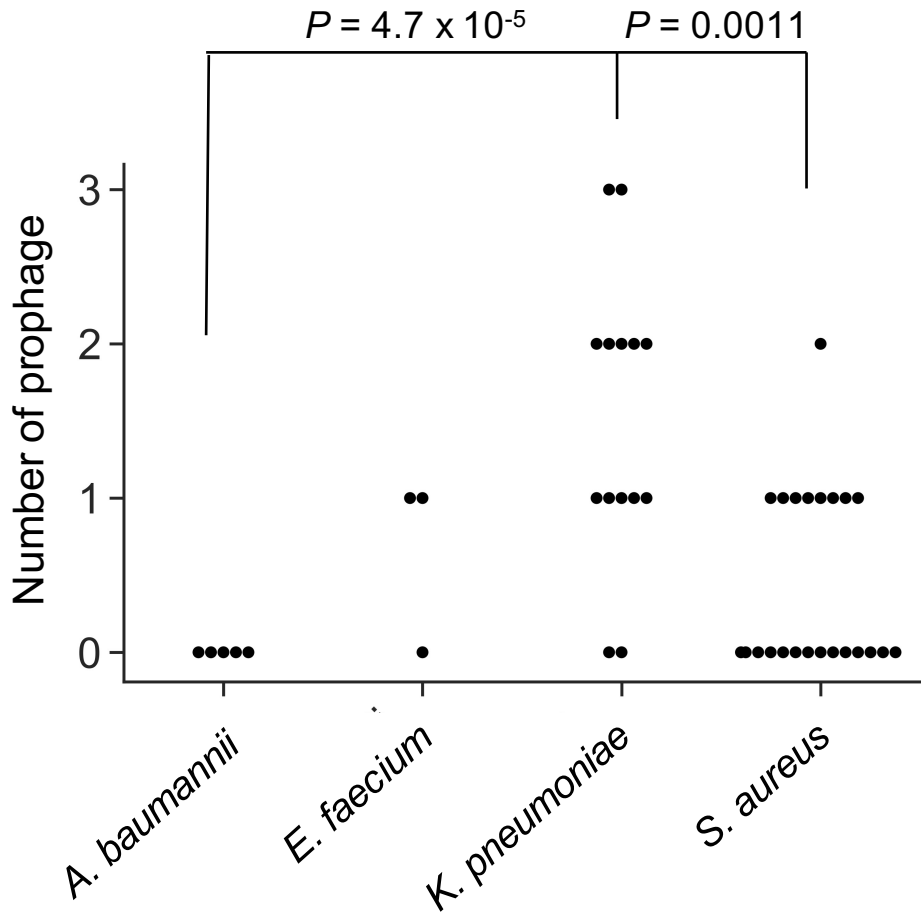

Supplement: FIG S2 [file msphere.00452-21-sf002.pdf]

Percentage (%)

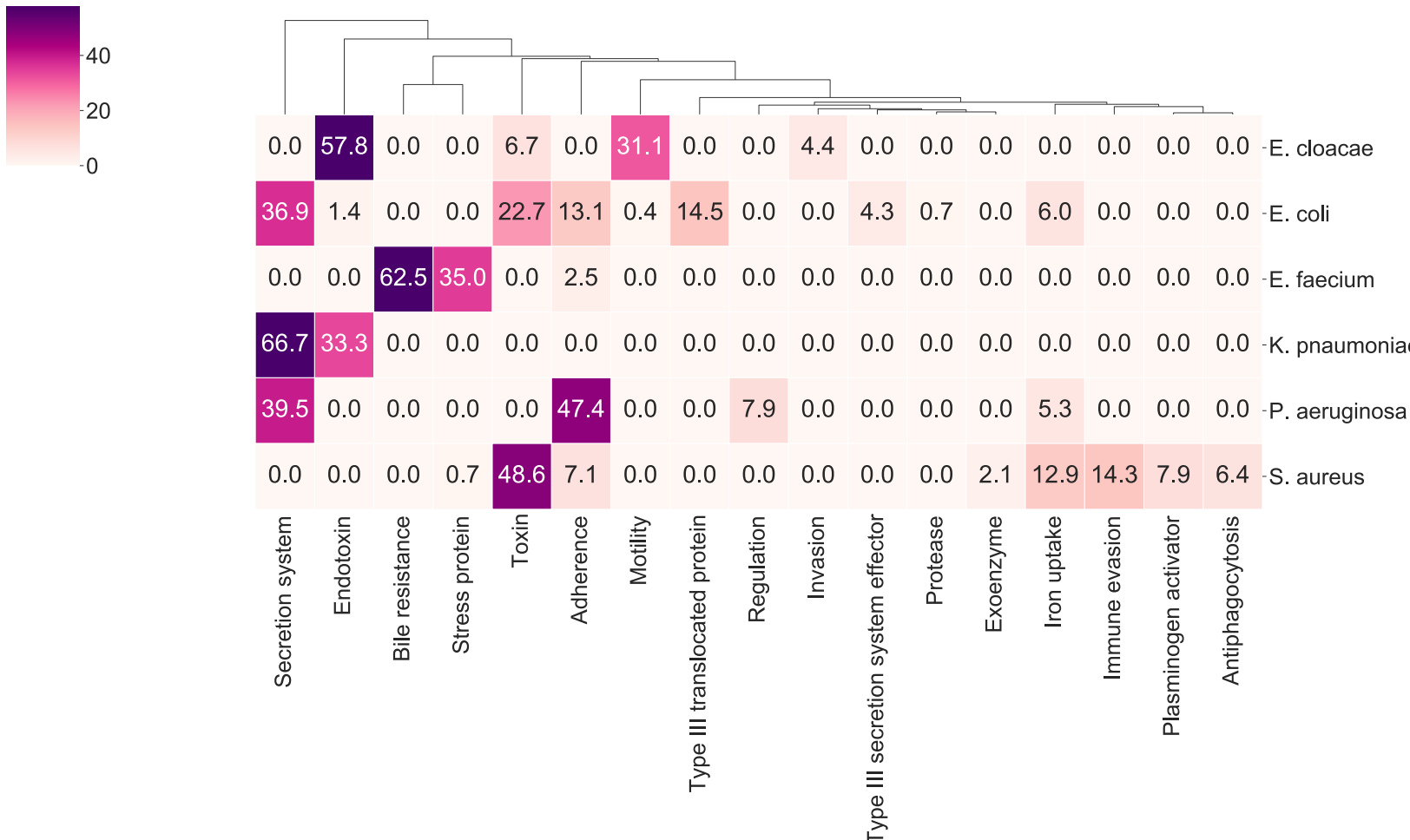

Supplement: FIG S4 [file msphere.00452-21-sf004.pdf]
