# Supplementary material for: Distribution of Antimicrobial Resistance and Virulence Genes within the Prophage-Associated Regions in Nosocomial Pathogens
Source: mSphere. 2021 Jul 7;6(4):e00452-21. doi: 10.1128/mSphere.00452-21 (PMC8386436; doi:10.1128/mSphere.00452-21)
Supplement: FIG S3 [file msphere.00452-21-sf003.pdf]

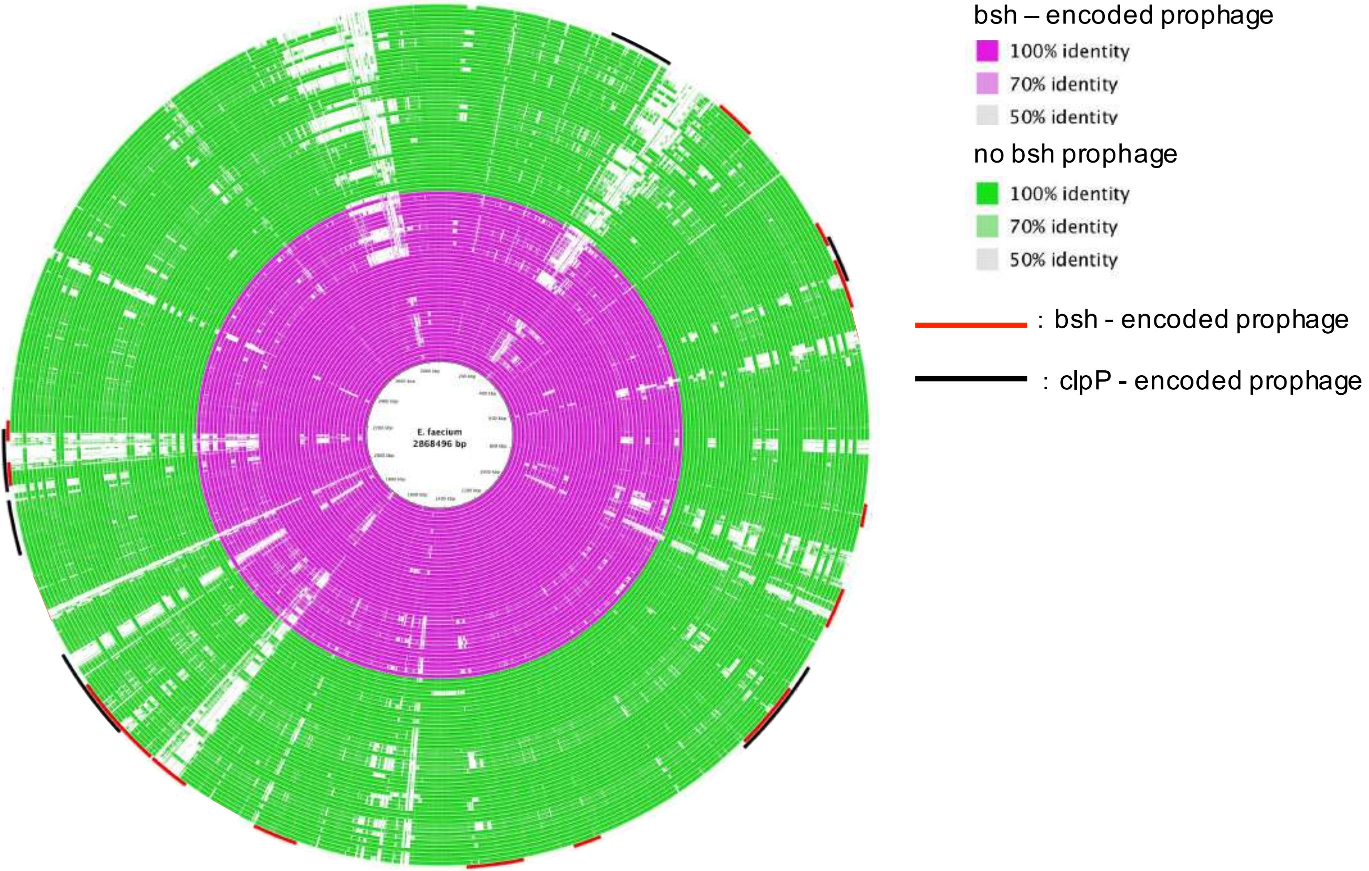

E. faecium
2868496 bp
bsh – encoded prophage
100% identity
70% identity
50% identity
no bsh prophage
100% identity
70% identity
50% identity
: bsh - encoded prophage
: clpP - encoded prophage
